# Supplementary material for: Identification of a Novel Semi-Dominant Spotted-Leaf Mutant with Enhanced Resistance to Xanthomonas oryzae pv. oryzae in Rice
Source: Int J Mol Sci. 2018 Nov 27;19(12):3766. doi: 10.3390/ijms19123766 (PMC6321207; doi:10.3390/ijms19123766)
Supplement: Supplementary file 1 [file ijms-19-03766-s001.zip › ijm-383098-sup.docx]

**Table S1.** The primers used in this study

| Primer | Forward sequence (5’-3’) | Reverse sequence (5’-3’) |
| --- | --- | --- |
| RM5961 | GATCAGCAGTGGACGATTCACC | TCTCCTGTATGCTCCTCCTCACC |
| RM26908 | TCGTCGTCGTCTTCCTCCTCTCC | GCGTGGAAGGTGAGGTAGGTCTGG |
| INDEL12 | ATGCGTGTACTTAAACAGATG | TGTCGATCTTGATAGTTTAG |
| INDEL33 | GATGAAGCTGGACGTCGGTG | CGAATCACGGACACCAGCG |
| RM5961 | GATCAGCAGTGGACGATTCACC | TCTCCTGTATGCTCCTCCTCACC |
| OsMC1 | GCTTCATCAAGGCGGTGGAGTG | AAGTTGGCGACCTTGCGGATG |
| OsMC2 | CGACCCGTACAGGGTGCCGA | GCACAGCGCCTCGTCGTAGC |
| OsMC3 | GGCTCCTTCGTCCGCAAGAT | CACAGGAGAAACGGTTTCCTGT |
| OsMC4 | TCGACGTTCGTGGAGATGCTC | ATTCACGAGCCGCCTGATCTT |
| OsMC5 | GTGCCAGACCGACCAGACAT | CCGCTCTTCTCCGACAGGAT |
| OsMC6 | CCACACCGCAGGGTTCTTCAT | GTCCAGGCTGCTGAGTGTATCC |
| OsMC7 | ATACAGACCGTGCTGGCGTC | AGGAATGGCGTCTCGGCGTT |
| OsMC8 | TCCGGCAAGTGCCTCGTAAC | CAATGCGGTCGGTCACAGGAT |
| OsPR1a | GGAAGTACGGCGAGAACATC | TGGTCGTACCACTGCTTCTC |
| OsPR1b | AGAACTACGCCAGCCAGAGAAG | TTCTCGCCAAGGTTGTTCCG |
| OsPR3 | CGTGTCTGTGGAGAGCGTGGTC | TCGTCGTTGGTGCGGTCATTGG |
| OsPR4 | AGTATGGATGGACCGCCTTCTGT | CTCGCAATTATTGTCGCACCTGTTC |
| OsPBZ1 | CCCTGCCGAATACGCCTAA | CTCAAACGCCACGAGAATTTG |
| OsPAL3 | CGCTGAGGCGTTTAAGATTG | GGCAAGGACAGCAAGAATG |
| OsPAL6 | AGATTGAGGTCATCCGTG | GAACATGAGCTTACCGATC |
| OsCHS1 | GACTACCCGGACTACTACTTCA | CTTCCTGATCTGCGACTTG |
| OsEDS1 | CATTCCAAGAACGAGGACACTG | CAAGACTCAAGGCTAGAACCGA |
| OsJAR1 | CTTCATTGACGCAGGCTACAC | GCACCACCAAGGCTTAGGA |
| OsAOS2 | CCCTAGCGTTGACAACAAGCA | CGGAGGTTGAAGCTTTGGTGA |
| OsWRKY45 | TTCCTTGTTGATGTGTCGTCTCA | CCCCCAGCTCATAATCAAGAAC |
| OsJAZ6 | GGACATGCCGATCGCGAGGAA | GCGCGAGTGCATGTGTCCAA |
| OsJAmyb | CCGAGCATGGTGACTAGCTCATCTT | CCTTGCACCCAACCGTTAAGCTGTT |
| OsPAD4 | CCAACATGTACCGCATCAAG | GGTTGTTTCGGTGGTAGTGG |
| Actin | AGGCTCCTCTCAACCCCAAG | TTTCCTGGTCATAGTCCAGG |
